# Importance of salt fingering for new nitrogen supply in the oligotrophic ocean

**DOI:** 10.1038/ncomms9002

**Published:** 2015-09-09

**Authors:** B. Fernández-Castro, B. Mouriño-Carballido, E. Marañón, P. Chouciño, J. Gago, T. Ramírez, M. Vidal, A. Bode, D. Blasco, S.-J. Royer, M. Estrada, R. Simó

**Affiliations:** 1 Departamento de Ecoloxía e Bioloxía Animal, Universidade de Vigo, 36310 Vigo, Spain; 2 Instituto Español de Oceanografía (IEO), Centro Oceanográfico de Vigo, Apdo. 1552, 36390 Vigo, Spain; 3 Instituto Español de Oceanografía (IEO), Centro Oceanográfico de Málaga, Puerto Pesquero s/n, Apdo. 285, 29640 Fuengirola (Málaga), Spain; 4 Departament d'Ecologia, Universitat de Barcelona, A. Diagonal 643, 08028 Barcelona, Spain; 5 Instituto Español de Oceanografía (IEO), Centro Oceanográfico de A Coruña, Apdo. 130, 15080 A Coruña, Spain; 6 Institut de Ciències del Mar, CSIC, Pg. Marítim de la Barceloneta, 37-49, 08003 Barcelona, Spain

## Abstract

The input of new nitrogen into the euphotic zone constrains the export of organic carbon to the deep ocean and thereby the biologically mediated long-term CO_2_ exchange between the ocean and atmosphere. In low-latitude open-ocean regions, turbulence-driven nitrate diffusion from the ocean's interior and biological fixation of atmospheric N_2_ are the main sources of new nitrogen for phytoplankton productivity. With measurements across the tropical and subtropical Atlantic, Pacific and Indian oceans, we show that nitrate diffusion (171±190 μmol m^−2^ d^−1^) dominates over N_2_ fixation (9.0±9.4 μmol m^−2^ d^−1^) at the time of sampling. Nitrate diffusion mediated by salt fingers is responsible for ca. 20% of the new nitrogen supply in several provinces of the Atlantic and Indian Oceans. Our results indicate that salt finger diffusion should be considered in present and future ocean nitrogen budgets, as it could supply globally 0.23–1.00 Tmol N yr^−1^ to the euphotic zone.

## Abstract (web summary)

The relative contribution of nitrogen fixation and nitrogen diffusion to marine biomes is presently debated. Here, the authors evaluate the contribution of these pathways across the tropics and subtropics of the global ocean and show that nitrogen diffusion, reinforced by salt fingers, is the dominant process.

## Graphical abstract

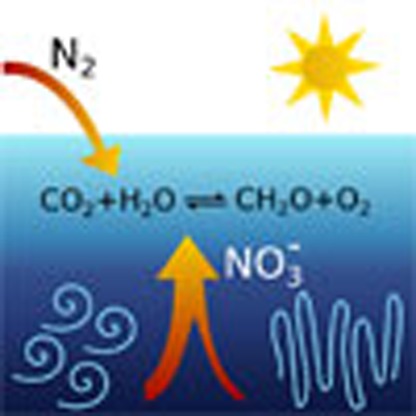

The concept of new production, as opposed to regenerated production, has been instrumental in understanding and modelling carbon export in the ocean^1^. This concept indicates that biological production susceptible of being exported outside the euphotic zone must be in balance with the input of new nutrients (as opposed to nutrient recycling) into the euphotic zone^1^. Turbulent diffusion across the nitracline has been traditionally considered the dominant source for new nitrogen to the surface ocean. However, recent studies indicate that biological fixation of atmospheric N_2_ by microbial diazotrophs could equal or even exceed nitrate diffusion as a mechanism for new nitrogen supply in the subtropical gyres^2^,^3^,^4^. These vast biomes are responsible for approximately 30% of the global carbon export to the deep ocean^5^,^6^. Below the mixed layer, turbulent diffusivity is due to mechanical processes, such as shear instabilities and internal waves, and also due to double-diffusive processes including salt fingers^7^. These develop in the tropical and subtropical central oceans, where warm and salty layers overlie cooler and fresher waters^8^. Because salt fingers mix dissolved substances more efficiently than mechanical turbulence^9^, this phenomenon could have important implications for the transport of nutrients and phytoplankton growth^10^. The first attempt to quantify the relevance of salt finger mixing to new production reported a sixfold increase in nitrate diffusive fluxes^11^. Despite the subsequent development of more accurate models to estimate salt finger diffusivity from microstructure measurements^12^, simultaneous estimates of the magnitudes of nitrate diffusion and N_2_ fixation have so far overlooked the contribution of this process^2^,^3^,^4^,^13^.

Between December 2010 and July 2011, during the Malaspina expedition, estimates of N_2_ fixation rates and nitrate diffusive fluxes, due to mechanical turbulence and salt fingers, were obtained in 40 stations located in different biogeographical provinces^14^ of the tropical and subtropical Atlantic (NE Atlantic Subtropical Gyral (NASE), North Atlantic Tropical Gyral (NATR), Western Tropical Atlantic (WTRA), South Atlantic Gyral (SATL) and Caribbean (CARB)), Indian (Indian South Subtropical Gyre (ISSG)) and Pacific oceans (South Pacific Subtropical Gyre (SPSG), Pacific Equatorial Divergence (PEQD), North Pacific Equatorial Countercurrent (PNEC) and North Pacific Tropical Gyre (NPTG)) (see Methods). Four additional stations were sampled along the coast of south Australia (South Subtropical Convergence (SSTC), East Australia Coastal (AUSE) and Australia-Indonesia Coastal (AUSW)). These stations were not considered for computing the global averages provided in the text, as they were located very close to the coast and therefore they are not representative of typical open-ocean conditions. N_2_ fixation was measured following the ^15^N_2_ bubble injection uptake technique described by Montoya *et al.*^15^, whereas vertical diffusivity was derived from shear and temperature microstructure observations, and the St Laurent and Schmitt^12^ model (see Methods). We show that, on average, nitrate diffusion dominated over N_2_ fixation, and that diffusion mediated by salt fingers was responsible for ca. 20% of the new nitrogen supply in several tropical and subtropical provinces of the north and south Atlantic, and the Indian Ocean.

## Results

### Nitrate diffusive fluxes

**Figure 1:**
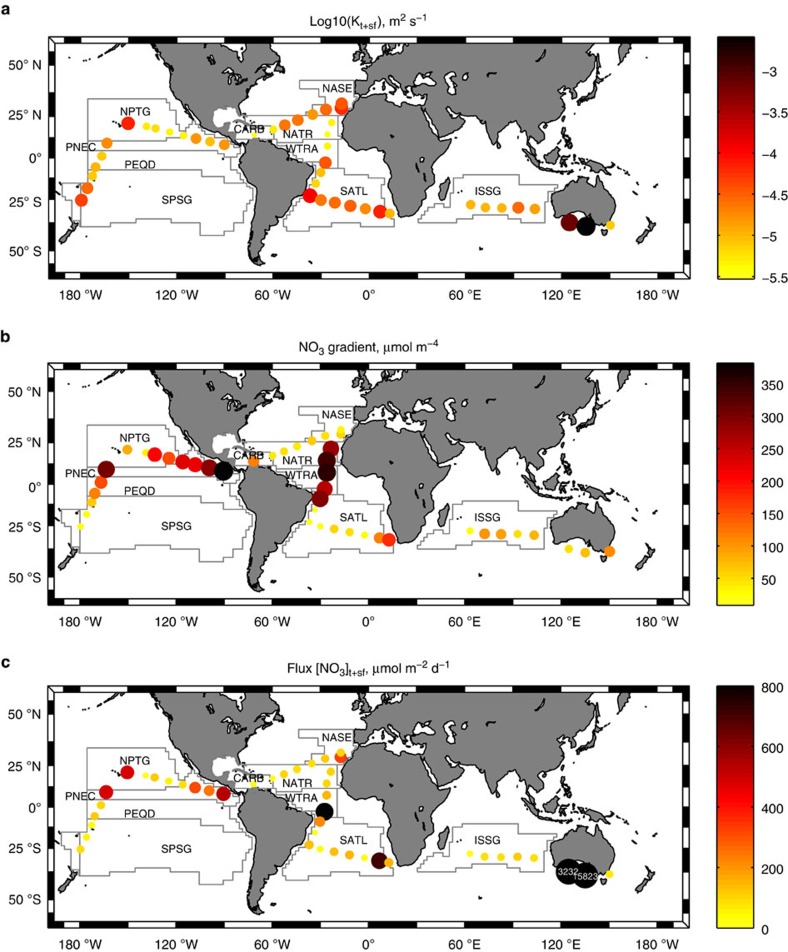
Nitrate diffusive fluxes during the Malaspina expedition. (**a**) Averaged vertical diffusivity including the effect of salt fingers plus mechanical turbulence (*K*_t+sf_), (**b**) nitrate gradient, and (**c**) nitrate diffusive fluxes across the nutricline. White numbers in black bubbles indicate values above the range shown in the colour bar. Main tropical and subtropical biogeographical provinces^14^ crossed during the expedition are shown: NASE (NE Atlantic Subtropical Gyral), NATR (North Atlantic Tropical Gyral), WTRA (Western Tropical Atlantic), SATL (South Atlantic Gyral) and CARB (Caribbean) in the Atlantic; ISSG (Indian South Subtropical Gyre) in the Indian; and SPSG (South Pacific Subtropical Gyre), PEQD (Pacific Equatorial Divergence), PNEC (North Pacific Equatorial Countercurrent) and NPTG (North Pacific Tropical Gyre) in the Pacific Oceans. Three other stations sampled along the coastal regions of Australia (SSTC, South Subropical Convergence, AUSE, East Australia Coastal and AUSW, Australia-Indonesia Coastal) are also included.

Vertical diffusivity by mechanical turbulence and salt fingers computed during the expedition ranged between 0.0296 × 10^−4^ and 25.6 × 10^−4^ m^2^ s^−1^, with the highest values observed near the southern coast of Australia (Fig. 1 and Supplementary Table 1). Nitrate gradients were in general >100 μmol m^−4^ in the tropical provinces, some of them influenced by the equatorial or the Costa Rica Dome upwelling (NATR, WTRA, PEQD and PNEC), and lower than this value in the subtropics (Table 1). Nitrate diffusive fluxes, computed as the product of vertical diffusivity and the nitrate gradient (see Methods), mostly ranged between 5.4 and 846.5 μmol m^−2^ d^−1^ (Supplementary Table 1) (global average 171±190 (±s.d.) μmol m^−2^ d^−1^). The regional variability of these fluxes was mainly driven by nitrate concentration gradients, as they were generally higher in the tropical Atlantic and Pacific (ranging 99.9–495.4 μmol m^−2^ d^−1^ in NATR, WTRA, PEQD and PNEC), compared to the subtropical and Caribbean provinces (25.6–177.7 μmol m^−2^ d^−1^ in NASE, SATL, CARB, ISSG and SPSG) (Table 1). The only exception to this general pattern were the higher values observed at two stations sampled near south Australia, due to the enhanced diffusivity.

Salt finger diffusion was negligible in the Pacific, where favourable stratification conditions for their formation were scarce^16^, and relevant in the Atlantic provinces SATL (0.17±0.35 × 10^−4^ m^2^ s^−1^), NASE (0.07±0.05 × 10^−4^ m^2^ s^−1^) and WTRA (0.07±0.11 × 10^−4^ m^2^ s^−1^), and the Indian ISSG (0.05±0.09 × 10^−4^ m^2^ s^−1^, see Table 1). The averaged nitrate diffusive flux due to salt fingers computed using all the open-ocean stations sampled during the expedition was 24±57 μmol m^−2^ d^−1^. However, stratification conditions favourable for salt fingers were only found in 21 stations. The average flux using only these stations was 46±76 μmol m^−2^ d^−1^. The higher nitrate fluxes due to salt finger mixing were computed for the tropical Atlantic WTRA (162.8±239.1 μmol m^−2^ d^−1^), followed by the Atlantic and Indian subtropic provinces NASE, SATL and ISSG (20.5–34.9 μmol m^−2^ d^−1^).

### N_2_ fixation and diazotrophic microplankton

**Figure 2:**
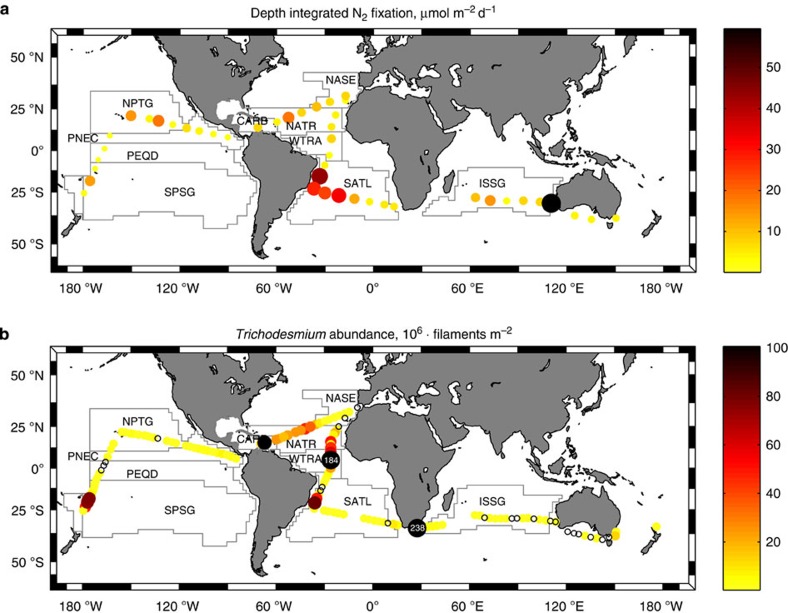
N_2_ fixation and *Trichodesmium* spp. abundance during the Malaspina expedition. (**a**) Photic layer depth-integrated N_2_ fixation rates and (**b**) *Trichodesmium* abundance estimated from vertical tows of a microplankton net in the upper 200 m (see Methods). White numbers in black bubbles indicate values above the range shown in the colour bar. White circles indicate stations where the organisms were not detected. Main tropical and subtropical biogeographical provinces^14^ crossed during the expedition are shown: NASE (NE Atlantic Subtropical Gyral), NATR (North Atlantic Tropical Gyral), WTRA (Western Tropical Atlantic), SATL (South Atlantic Gyral) and CARB (Caribbean) in the Atlantic; ISSG (Indian South Subtropical Gyre) in the Indian; and SPSG (South Pacific Subtropical Gyre), PEQD (Pacific Equatorial Divergence), PNEC (North Pacific Equatorial Countercurrent) and NPTG (North Pacific Tropical Gyre) in the Pacific Oceans. Four other stations sampled for N_2_ fixation along the coastal regions of Australia (SSTC, South Subropical Convergence, AUSE, East Australia Coastal and AUSW, Australia-Indonesia Coastal) are also included.

**Figure 3:**
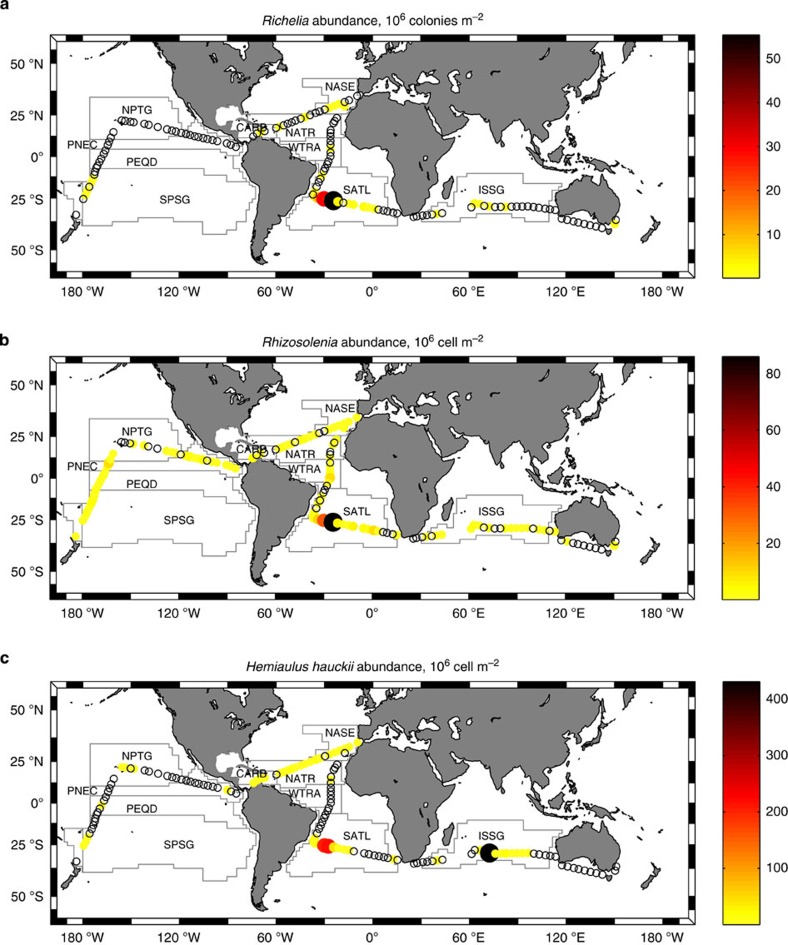
Diazotrophic microplankton abundance during the Malaspina expedition. Photic layer depth-integrated abundance of (**a**) *Richelia colonies*, (**b**) *Rhizosolenia* spp. and (**c**) *Hemiliaulus hauckii* spp. collected during the Malaspina expedition. White circles indicate stations where the organisms were not detected. Main tropical and subtropical biogeographical provinces^14^ crossed during the expedition are shown: NASE (NE Atlantic Subtropical Gyral), NATR (North Atlantic Tropical Gyral), WTRA (Western Tropical Atlantic), SATL (South Atlantic Gyral) and CARB (Caribbean) in the Atlantic; ISSG (Indian South Subtropical Gyre) in the Indian; and SPSG (South Pacific Subtropical Gyre), PEQD (Pacific Equatorial Divergence), PNEC (North Pacific Equatorial Countercurrent) and NPTG (North Pacific Tropical Gyre) in the Pacific Oceans.

Photic layer depth-integrated N_2_ fixation rates ranged between 0.031 and 59.5 μmol m^−2^ d^−1^ (average 9.0±9.4 μmol m^−2^ d^−1^), but in most cases were below 10 μmol m^−2^ d^−1^ (Fig. 2 and Supplementary Table 1). The maximum rate of 59.5 μmol m^−2^ d^−1^ was measured in the eastern Indian ocean, and the highest province-averaged rate corresponded to the south Atlantic SATL (17.8±14.8 μmol m^−2^ d^−1^) (Table 1). The abundance of the colony-forming diazotrophic cyanobacterium *Trichodesmium* spp. was higher (>40 × 10^6^ filaments m^−2^) in the Atlantic WTRA and CARB, followed by the Atlantic NATR and the Pacific SPSG (>20 × 10^6^ filaments m^−2^) (Table 2). The diazotrophic endosymbiont *Richelia intracellularis* and its hosts, the diatoms *Hemiaulus hauckii* and *Rhizosolenia* spp., were more abundant in the SATL (Fig. 3 and Table 2).

### Relative contributions to the new nitrogen supply

**Figure 4:**
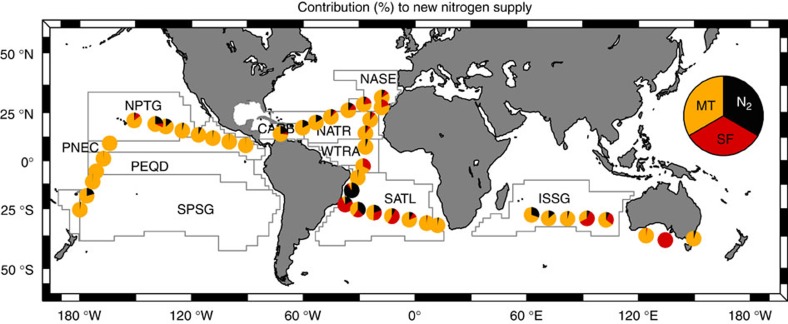
Relative contribution of nitrate diffusive fluxes and N_2_ fixation to the new nitrogen supply during the Malaspina expedition. Relative contribution (%) of nitrate diffusive fluxes due to mechanical turbulence (MT, orange) and salt fingers (SF, red), and N_2_ fixation (N_2_, black) to the new nitrogen supply, considered as the sum of these three processes. Main tropical and subtropical biogeographical provinces^14^ crossed during the expedition are shown: NASE (NE Atlantic Subtropical Gyral), NATR (North Atlantic Tropical Gyral), WTRA (Western Tropical Atlantic), SATL (South Atlantic Gyral) and CARB (Caribbean) in the Atlantic; ISSG (Indian South Subtropical Gyre) in the Indian; and SPSG (South Pacific Subtropical Gyre), PEQD (Pacific Equatorial Divergence), PNEC (North Pacific Equatorial Countercurrent) and NPTG (North Pacific Tropical Gyre) in the Pacific Oceans. Three other stations sampled along the coastal regions of Australia (SSTC, South Subropical Convergence, AUSE, East Australia Coastal and AUSW, Australia-Indonesia Coastal) are shown in the map.

The relative contribution of nitrate diffusive fluxes (due both to mechanical turbulence and salt fingers) and N_2_ fixation to the new nitrogen supply, here considered as the total amount of new nitrogen supplied by these processes, is reported in Fig. 4, Table 1 and Supplementary Table 1. It is important to note that several other mechanisms, such as mesoscale and submesoscale turbulence, lateral transport, atmospheric deposition and the more complex three-dimensional dynamics, today recognized as important contributors to the supply of new nitrogen^17^,^18^,^19^,^20^, were not considered in our study. On average N_2_ fixation represented 10±15% of the new nitrogen supply. This process represented >50% of the new nitrogen supply only in one station, located in the western SATL, where relatively high N_2_ fixation rates coincided with low diffusive fluxes. Other than that, the higher contributions of N_2_ fixation occurred in the Atlantic provinces SATL (21%) and CARB (19%) (Table 1). The global averaged contribution of salt finger mixing was 11±18%. Although the averaged contribution of N_2_ fixation and salt finger mixing to the new nitrogen supply was very similar, both processes showed a different regional distribution. The contribution of salt fingers was >50% only in four stations, located in the Atlantic SATL, the Indian ISSG and the coastal south Australia (Supplementary Table 1). In terms of provinces, higher averaged contributions were computed for the Atlantic SATL (24%), WTRA (19%) and NASE (19%), and the Indian ISSG (18%), whereas the contribution in the Pacific provinces was <5% (Table 1).

## Discussion

Nitrate diffusive fluxes computed during the Malaspina expedition are in the range of previously reported values for open-ocean (34–850 μmol m^−2^ d^−1^)^3^,^4^,^21^,^22^ and regions under the influence of equatorial upwelling (1,300 μmol m^−2^ d^−1^)^23^. Despite the relevant contribution of salt fingers to nitrate diffusion described for our data in several Atlantic and Indian provinces, our estimates are far below the five- to sixfold increase factor, with respect to mechanical turbulence, reported by Hamilton *et al.*^11^ and Dietze *et al.*^24^. Differently to our study, Hamilton *et al.* used the conservation equation of turbulent kinetic energy to derive salt finger mixing efficiency, by assuming that there is no mechanical generation of turbulence. However, more recent studies demonstrated that in the ocean mechanical turbulence could disrupt the effect of salt fingers, causing lower mixing efficiencies than those predicted by the Hamilton *et al.* model^12^ (see Methods). On the other hand, Dietze *et al.*^24^ computed nitrate diffusive fluxes using two parameterizations for internal waves^25^ and salt finger diffusivity^26^ based on conductivity–temperature–depth (CTD) and current velocity data. Simultaneous microstructure-derived and parameterized diffusivity estimates are scarce; for this reason, and also because of the different nature of both estimates, the comparison is difficult to make. In a previous study we compared microstructure-derived and K-profile parameterized (KPP) diffusivity estimates performed during the Malaspina expedition^16^. In general, the KPP showed a good agreement with diffusivity estimates derived from microstructure observations. However, the relative contribution of salt finger mixing derived from the KPP was higher compared to the estimates derived from microstructure observations. Our estimates are in good agreement with recent studies using the same model to estimate the contribution of salt finger mixing^21^; therefore discrepancy with previous studies is probably due to the different approaches used.

Excluding the Atlantic NASE and SATL provinces, N_2_ fixation rates measured during the Malaspina expedition fall within the lower edge of previous estimates recently compilated by Luo *et al.*^27^ (Table 1). Some of the previously described spots of enhanced N_2_ fixation, such as the Caribbean and the tropical North Atlantic^2^,^28^,^29^, were not detected here. This was probably due to the low spatial resolution of our sampling for N_2_ fixation rates (ca. 1,000 km), as these spots were indeed tracked by the higher spatial resolution of the *Trichodesmium* abundance measurements. The fact that *Trichodesmium* abundance did not show any statistically significant correlation with N_2_ fixation rates (*r*=−0.059, *P*=0.71) points to the contribution of other groups of diazotrophs. High abundances of the diatoms *Hemiaulus hauckii* and *Rhisozolenia* hosting the diazotrophic symbiont *Richelia intracelularis* found in the western SATL, could possibly be related to the enhanced N_2_ fixation rates determined in this region (Fig. 3). Elevated N_2_ fixation rates as the result of this symbiosis have been previously reported in the Amazon river plume region^30^. Moreover, the symbiotic cyanobacterium UCYN-A hosted by a prymnesiophyte was also detected during the Malaspina expedition in regions of enhanced N_2_ fixation rates, such as the western SATL and the eastern Indian ocean^31^. Indeed, the rates we measured in tropical and subtropical regions are in good agreement with the background N_2_ fixation rates of 8–16 μmol m^−2^ d^−1^ reported for unicellular diazotrophs in the north Atlantic^4^,^28^.

It has recently been reported that the ^15^N_2_ bubble injection technique underestimates N_2_ fixation rates because the injected bubble does not attain equilibrium with the water^32^,^33^. According to Mohr *et al.*^32^ the underestimation is significantly reduced when, similar to the methodology used during the Malaspina expedition (see Methods), 24-h incubations are used. However, recent studies suggest that the underestimation could persist for 24-h incubation periods^34^. Although there is a growing interest among the scientific community to establish a protocol to solve this problem, so far no consensus has been attained. In any case, the underestimation problem would also affect most of the previously published N_2_ fixation rates^27^.

**Figure 5:**
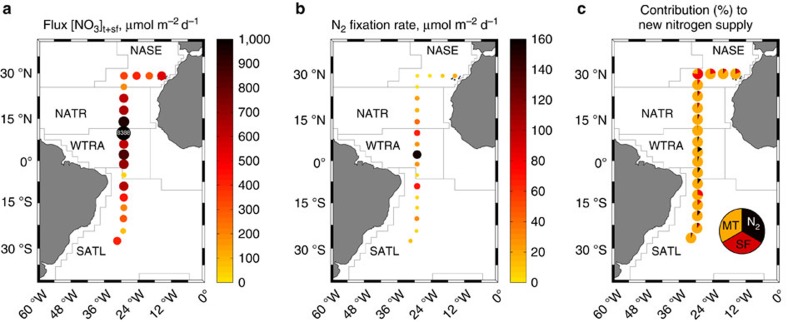
N_2_ fixation and nitrate diffusive fluxes during the *TRYNITROP* cruise. (**a**) Nitrate diffusive fluxes computed across the nutricline, due to mechanical turbulence plus salt finger diffusivity, (**b**) photic layer depth integrated N_2_ fixation rates and (**c**) relative contribution (%) of mechanical turbulence (MT, orange), salt fingers (SF, red) and N_2_ fixation (N_2_, black) to the new nitrogen supply, considered as the sum of these three processes. White numbers in black bubbles indicate values above the range shown in the colour bar. Main biogeographical provinces^14^ crossed during the *TRYNITROP* cruise are shown in the map: NASE (NE Atlantic Subtropical Gyral), NATR (North Atlantic Tropical Gyral), WTRA (Western Tropical Atlantic) and SATL (South Atlantic Gyral).

To our knowledge only three studies have compared so far the relevance of nitrate diffusion and N_2_ fixation in the open ocean^2^,^3^,^4^, none of them including the contribution of salt fingers to mixing. In the tropical north Atlantic, Capone *et al.*^2^ estimated that N_2_ fixation by *Trichodesmium* spp. could equal or even exceed the vertical flux of nitrate into the euphotic zone as estimated by using a constant diffusivity. The use of a microstructure profiler along a meridional transect in the Atlantic (*TRYNITROP* cruise) revealed the importance of considering the variability in diffusivity^3^. These authors estimated a contribution of N_2_ fixation to the new nitrogen supply of 44±30%, 22±19% and 2±2% in the south subtropical, equatorial and north subtropical Atlantic, respectively. Finally, Painter *et al.*^4^ reported N_2_ fixation rates comparable to nitrate diffusive fluxes, also derived from a microstructure profiler, in the northeast subtropical Atlantic. The comparison of fluxes reported in different studies is sometimes problematic because nitrate diffusive fluxes are very sensitive to the depth interval chosen for the calculation. The nitrate fluxes reported during the *TRYNITROP* cruise in Mouriño Carballido *et al.*^3^ were computed across a 10-m layer centred at the bottom of the photic layer and they were interpreted as instantaneous. Here we chose to compute the fluxes across the upper nitracline, which results from biological consumption at the surface and therefore integrates processes occurring at relatively longer temporal scales. The recalculation of nitrate diffusive fluxes during the *TRYNITROP* cruise following exactly the same protocol reported here, in order to include mechanical turbulent mixing but also salt fingers (Fig. 5), shows a maximum contribution of salt finger mixing to new production in NASE (26±28%) and SATL (8.3±9.6%), higher than the contribution of N_2_ fixation (1.7±1.4% and 4.9±3.6%, respectively), and consistent with the results from the Malaspina expedition.

The data set collected during the Malaspina circumnavigation allowed us to compare, for the first time, the contribution of N_2_ fixation, mechanical turbulence and salt finger mixing to the supply of new nitrogen to surface euphotic waters in large regions of the open ocean. These data reveal that nitrate diffusion dominated over N_2_ fixation at the time of sampling, and highlight the importance of considering the effect of salt finger mixing in nitrogen budgets of the surface ocean. This process was an important source for new nitrogen, representing close to 20% of the new nitrogen supply in the Atlantic tropical and subtropical NASE, WTRA and SATL provinces, and the subtropical Indian ISSG (Table 1). Although salt finger mixing and N_2_ fixation showed different regional distribution, on average, the contribution of both processes as sources of new nitrogen supply was comparable. Albeit the Malaspina expedition provided a unique data set, this study has limited temporal and spatial resolution. In order to provide a global estimate of nitrate diffusive fluxes due to salt finger mixing, we combined data from the World Ocean Atlas 2009 ( http://www.nodc.noaa.gov/) and the KPP^35^ (Supplementary Fig. 1). The computed supply flux by this process (1.00±0.75 Tmol yr^−1^) is about fourfold higher than the global estimate derived from extrapolating the averaged salt finger flux estimated from the Malaspina observations (0.23 Tmol yr^−1^). These figures are within the range of global N_2_ fixation (0.36–11 Tmol yr^−1^)^36^ and atmospheric N deposition (2–6 Tmol yr^−1^)^37^ estimates, emphasizing the need to include salt finger mixing in present and future ocean nitrogen budgets.

## Methods

### Sampling

Field observations were carried out during the Malaspina circumnavigation expedition in the Atlantic, Pacific and Indian Oceans between December 2010 and July 2011 on board R/V *Hespérides* (see Supplementary Fig. 2). The cruise was divided into seven legs: leg 1 (14 December 2010, Cádiz–13 January 2011, Rio de Janeiro), leg 2 (17 January, Rio de Janeiro–6 February, Cape Town), leg 3 (11 February, Cape Town–13 March, Perth), leg 4 (17 March, Perth–30 March, Sydney), leg 5 (16 April, Auckland–8 May, Honolulu), leg 6 (13 May, Honolulu–10 June, Cartagena de Indias) and leg 7 (19 June, Cartagena de Indias–14 July, Cartagena). Leg 1 crossed the NASE, NATR, WTRA and SATL biogeographical provinces^14^. Leg 2 sampled a zonal transect across the SATL province, the last station being carried out in the Benguela Current Coastal (BENG). Leg 3 crossed the Indian ocean from west to east. The first three stations were carried out in the East Africa Coastal (EARF) province, whereas most of the stations sampled the ISSG province. Four provinces were sampled along the south Australian coast during leg 4: ISSG, AUSW, SSTC and AUSE provinces. During leg 5, the SPSG, PEQD, PNEC and NPTG provinces were sampled. Leg 6 crossed the NPTG and again the PNEC province. Finally, during leg 7 the CARB, NATR and NASE provinces were sampled.

CTD casts were carried out with a SBE911plus (Sea-Bird Electronics) probe attached to a rosette equipped with Niskin bottles. A Lowered Acoustic Doppler Current Profiler (LADCP) system was also mounted on the rosette. A microstructure turbulence profiler was deployed in 36 stations. Water from Niskin bottles was collected in 39 stations for nutrient analysis, in 44 stations for the determination of N_2_ fixation rates and in 136 stations for microphytoplankton abundance determination. Samples for the determination of *Trichodesmium* abundance from a plankton net were collected in 132 stations.

### Microstructure measurements

Measurements of microstructure shear and temperature used to infer dissipation rates of turbulent kinetic energy (*ɛ*) and thermal variance (*χ*) were conducted by using a microstructure turbulence profiler MSS^38^, down to a maximum depth of 300 m. Averaged diffusivity due to both mechanical turbulence and salt fingers (*K*_t+sf_) was modelled, according to St Laurent and Schmitt^12^, as the weighed sum of diffusivity due to turbulence (*K*_t_) and salt fingers (
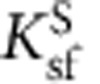
, where S stands for salt as we assumed that nitrate diffuses at the same rate as salinity): 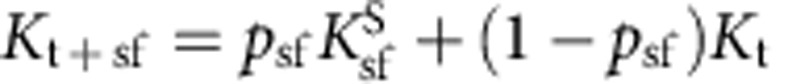, where *p*_sf_ is the weighting factor, corresponding to the fraction of bins where salt fingers are active. Favourable stratification for salt fingers was identified using the density ratio (*R*_*ρ*_=*α*∂_*z*_*T*/*β*∂_*z*_*S*, where *α* and *β* are the thermal expansion and salinity contraction coefficients, respectively). Although salt fingers are theoretically possible for *R*_*ρ*_>1, its contribution to mixing has been shown to be irrelevant for *R*_*ρ*_>2 (ref. 12). According to McDougall^39^ and Hamilton *et al.*^11^, who solved the turbulent kinetic energy equation for salt fingers, mixing efficiency for this process is expected to exceed the value of 0.2 for mechanical turbulence. Hence, we used two parameters to identify salt finger active bins: the density ratio (1<*R*_*ρ*_<2) and the observed mixing efficiency (Γ_Obs_>0.2) (calculated as Γ_Obs_=0.5*N*^2^*χ*/*ɛ*(∂_*z*_*T*)^2^, where *N* is the buoyancy frequency). Diffusivity for turbulence (*K*_t_) and salt finger (*K*_sf_^*T*^) bins was computed following the Osborn^40^, *K*_t_=*K*_*ɛ*_=〈0.2*ɛ*/*N*^2^〉, and the Osborn–Cox^41^ models, *K*_sf_^*T*^=*K*_*χ*_=〈0.5*χ*/(∂_*z*_*T*)^2^〉, respectively. The Osborn–Cox^41^ model applies for heat. For dissolved substances (i.e., nitrate) 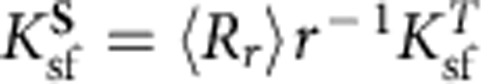, where *r*=0.4–0.7, according to the compilation of estimates carried out by St Laurent and Schmitt^12^, is the salt finger flux ratio. Here we set *r*=0.7 for coherence with the *K*-profile parameterization (see below). In the manuscript, salt finger diffusivity *K*_sf_ refers to the weighted contribution of this mechanism to total diffusivity: 
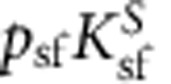
. Errors in turbulence plus salt fingers (*K*_t+sf_) and salt fingers diffusivity (*K*_sf_) were estimated as the standard deviation of 1,000 estimates obtained by bootstraping the input variables: *α*, *β*, *R*_*ρ*_, *K*_*ɛ*_ and *K*_*χ*_. In this way, *ɛ* and *χ* uncertainties were treated implicitly through *K*_*ɛ*_ and *K*_*χ*_. A 10% error estimate of the salt fingers flux ratio was added to the calculations. A sensitivity test was performed to verify that the computed diffusivity was not strongly dependent on the choice of the critical Γ value (data not shown).

During leg 3, when we sampled the Indian Ocean, and due to technical problems, no microstructure measurements were available, so diffusivity was estimated by using an adaptation of the KPP^35^ based on CTD, LADCP and meteorological data. The salt finger term included in the KPP was considered as *K*_sf_. Uncertainties of KPP diffusivity were calculated as the standard deviation of 1,000 averaged estimates resulting from bootstraping individual 10-m vertical resolution *K* values within the nitracline. A detailed description of the implementation of the KPP and the comparison with diffusivity derived from microstructure observations collected during the Malaspina expedition is given in Fernández-Castro *et al.*^16^.

### Nitrate diffusive fluxes

Nitrate diffusive fluxes across the nutricline were calculated following the Fick's law as


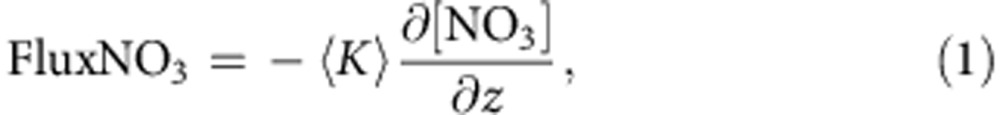


where 
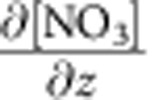
 is the nitrate gradient obtained by linearly fitting nitrate concentrations in the nitracline, and 〈*K*〉 is averaged diffusivity for the same depth range.

A total of 7–9 samples for nitrate (NO_3_)+nitrite (NO_2_) concentration in the upper 300 m were directly collected from the Niskin bottles in 20 ml acid-washed polyethylene vials. They were immediately analysed on board according to classical methods using the automated colorimetric technique^42^ on a segmented flow Skalar autoanalyser. For the nitracline region, relevant for this study, vertical resolution varied between 20 and 50 m. The nitracline was determined as a region of approximately maximum and constant gradient, usually extending to 50–100 m and including 4–6 nitrate data points. In those stations where nitrate concentrations were not available (see Supplementary Fig. 2) nitrate gradients were computed from the data included in the World Ocean Atlas 2009 (WOA09) database^43^. A good correspondence between nitrate gradients based on Malaspina observations and the WOA09 climatology was found (*r*=0.76, *P*<0.001, data not shown). Diffusive flux errors were calculated as the squared sum of the diffusivity error (see above) and the error of the slope resulting from the linear fit.

Nitrate diffusive fluxes due to mechanical turbulence and salt finger mixing during the *TRYNITROP* cruise, which sampled a meridional transect from 30°S to 30°N in the Atlantic Ocean in April–May 2008 (ref. 3), were calculated following exactly the same protocol as described for the Malaspina expedition.

### N_2_ fixation

Samples for the determination of N_2_ fixation rates were collected at the same stations where nitrate diffusive fluxes were computed. Additionally, samples were also collected at one station located at the Indian Australia coast (AUSW). Only those stations located in tropical and subtropical regions were used for computed global open-ocean averages reported in the text. N_2_ fixation was measured at four depths (surface, the depths where the photosynthetically active radiation was 20 and 10% of the surface value, and the deep chlorophyll maximum) following the ^15^N_2_ uptake technique described by Montoya *et al.*^15^. For each depth, one (three during leg 6) acid-washed, clear polycarbonate bottle (4 l in volume) was filled directly from the CTD rosette and supplemented with 8 ml of ^15^N_2_ (98 atom%; Sigma-Aldrich, lot -CX0937). Samples were incubated on deck at their original irradiance and temperature conditions during 24 h. After the incubation the whole volume was filtered through a 25-mm GF/F filter (Whatman). Afterwards, filters were dried at 40 °C for 24 h and then stored until pelletization in tin capsules. ^15^N atom % in particulate organic matter was measured with an elemental analyser combined with a continuous-flow stable isotope mass spectrometer (Flash-EA112+Deltaplus; ThermoFinnigan), using an acetanilide standard as reference. The equations given by Weiss^44^ and Montoya *et al.*^15^ were used to calculate the initial N_2_ concentration and N_2_ fixation rates, respectively.

### *Trichodesmium* spp. abundance

Plankton samples were collected by vertical tows of a microplankton net (40 μm mesh size) through the upper 200 m of the water column. Sampling was between 10:00 and 16:00 h GMT. Abundance of the diazotroph *Trichodesmium* spp. was estimated by counts of 50-ml aliquots of the sample from the microplankton net preserved in glutaraldehyde (25% final concentration) using a FlowCAM system (Fluid Imaging Technologies). Prior to analysis, the samples were screened with a 100-μm nylon mesh to prevent clogging of the FlowCAM cell. Results are reported as number of colonies (trichomes) per square meter.

### Diazotrophic microphytoplankton abundance

Samples for determining the abundance of diatom *Hemiliaulus hauckii* sp. and *Rhizosolenia* spp. hosts of the diazotrophic cyanobacteria *Richelia intracellularis*, and abundances of *Richelia intracellularis* colonies were collected at three depths from the surface to the deep chlorophyll maximum and fixed with 2% formalin–hexamine solution. Abundance was quantified using an inverted microscope, after 48-h sedimentation of 150 ml of sample in composite-settling chambers.

### Global supply of nitrate due to salt finger diffusivity

A gross estimate of vertical nitrate fluxes due to salt finger diffusivity between 40°S and 40°N was computed by using data from the WOA09. Vertical nitrate gradients were computed in the upper 250 m, and the nitracline was determined as the depth of maximum gradient. Temperature^45^ and salinity^46^ fields were used to compute the density ratio (*R*_*ρ*_). Vertical diffusivity due to salt finger mixing was calculated according to the KPP^35^ as a function of *R*_*ρ*_:


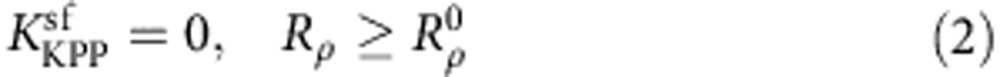



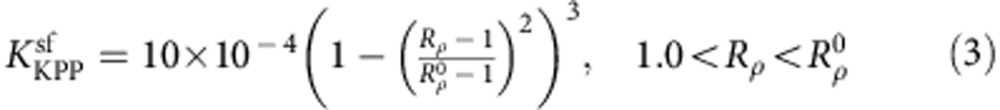


where 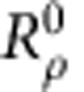
=1.9. Nitrate vertical fluxes due to salt finger mixing were calculated using equation (1). Errors were calculated by propagating standard errors of the temperature, salinity and nitrate fields.

A second global estimate was calculated by extrapolating the averaged nitrate diffusive flux due to salt fingers (46±76 μmol m^−2^ d^−1^), computed by using only the 21 stations where stratification conditions were favourable for salt finger formation (1<*R*_*ρ*_<2) during the Malaspina expedition, to the global surface area where this condition was accomplished in the nutricline (ca. 1.3 × 10^7^ km^2^), according to the WOA09 temperature and salinity fields.

## Additional information

**How to cite this article:** Fernández-Castro, B. *et al.* Importance of salt fingering for new nitrogen supply in the oligotrophic ocean. *Nat. Commun.* 6:8002 doi: 10.1038/ncomms9002 (2015).

## Supplementary Material

Supplementary Information Supplementary Figures 1-2 and Supplementary Table 1

## Figures and Tables

**Table 1:** Contribution of N_2_ fixation and nitrate diffusive fluxes to the new nitrogen supply during the Malaspina expedition.

**Prov.**	***n***_**T**_ **(*****n***_**P**_**)**	**N**_**2**_ **fix.μmol m**^−2^ **d**^−1^	**∂** **[NO**_**3**_**]/∂*****z*****μmol m**^−4^	***K***_**t+sf**_**10**^−4^** m**^2^ **s**^−1^****	**Flux**_**t+sf**_ **NO**_**3**_**μmol m**^−2^ **d**^−1^	***K***_**sf**_**10**^−4^** m**^2^ **s**^−1^****	**Flux**_**sf**_ **NO**_**3**_**μmol m**^−2^ **d**^−1^	**% N**_**2**_ **fix.**	**% sf.**	**N**_**2**_ **fix** **Luo** ***et al***.^27^**μmol m**^−2^ **d**^−1^
NASE	4 (0)	6.9±3.1	−52±11	0.35±0.21	160.0±118.4	0.07±0.05	33.8±29.4	6.0±4.2	19.2±4.0	4.1±1.2
NATR	4 (0)	8.7±6.6	−177±155	0.14±0.12	99.9±12.6	0.01±0.01	6.6±5.3	8.0±5.6	6.1±3.9	390.4±141.1
WTRA	2 (0)	5.5±3.9	−309±88	0.21±0.23	495.4±502.9	0.07±0.11	162.8±239.1	2.9±3.6	19.2±27.1	199.9±74.2
SATL	9 (1)	17.8±14.8	−85±95	0.35±0.37	177.7±214.9	0.17±0.35	34.9±46.5	20.6±28.1	24.1±27.6	12.5±4.3
CARB	2 (0)	6.1±2.4	−80±56	0.04±0.02	25.6±7.4	0.00±0.00	1.7±2.4	18.8±1.9	4.3±6.1	756.7±296.8
ISSG	5 (5)	8.7±4.0	−69±32	0.15±0.09	80.5±37.8	0.05±0.09	20.5±35.7	12.4±10.6	17.6±26.9	–±–
SPSG	3 (0)	5.3±7.8	−37±25	0.27±0.19	63.4±23.9	0.00±0.00	0.0±0.0	7.7±11.5	0.0±0.0	56.6±6.5
PEQD	2 (0)	0.3±0.4	−137±29	0.10±0.02	112.2±16.0	0.00±0.00	0.0±0.0	0.2±0.3	0.0±0.0	75.8±26.5
PNEC	4 (0)	2.0±1.9	−300±70	0.15±0.04	397.2±156.0	0.00±0.00	0.0±0.0	0.6±0.6	0.0±0.0	22.6±5.1
NPTG	5 (0)	9.3±6.6	−142±84	0.18±0.28	153.6±193.5	0.02±0.04	13.3±30.0	10.2±8.7	4.5±6.3	177.9±44.2

Averaged photic layer depth-integrated N_2_ fixation rates (N_2_ fix.), nitrate gradient (∂ [NO_3_]/∂*z*), vertical diffusivity (*K*_t+sf_) and nitrate diffusive fluxes due to salt fingers plus mechanical turbulence (Flux_t+sf_ NO_3_), vertical diffusivity (*K*_sf_) and nitrate diffusive fluxes due to salt fingers (Flux_sf_ NO_3_), and relative contribution of N_2_ fixation (% N_2_ fix.) and salt fingers (% sf.) to the new nitrogen supply computed for the tropical and subtropical biogeographical provinces crossed during the Malaspina expedition (Prov.): NASE (NE Atlantic Subtropical Gyral), NATR (North Atlantic Tropical Gyral), WTRA (Western Tropical Atlantic), SATL (South Atlantic Gyral), CARB (Caribbean), SPSG (South Pacific Subtropical Gyre), PEQD (Pacific Equatorial Divergence), PNEC (North Pacific Equatorial Countercurrent) and NPTG (North Pacific Tropical Gyre). Averaged N_2_ fixation rates compilated by Luo *et al.*^27^ at each province are also included. *n*_T_ is the number of stations where N_2_ fixation rates and nitrate diffusive fluxes were computed for each province, and *n*_P_ corresponds to the stations where KPP diffusivities were used. Errors correspond to standard deviations of the values shown in Supplementary Table 1. Uncertainties inherent to vertical diffusivity, NO_3_ gradients and nitrate diffusive flux calculations were quadratically added to standard deviations.

**Table 2:** Diazotrophic microplankton abundance during the Malaspina expedition.

**Prov**	***n***_**Trich**_	***Trichodesmium***	***n***_**dia**_	***Richelia***	***Rhizosolenia***	***Hemiaulus Hauckii***
		**10**^6^ **trichomes m**^−2^		**10**^6^ **colonies m**^−2^	**10**^6^ **cells m**^−2^	**10**^6^ **cells m**^−2^
NASE	11	3.20±5.64	10	0.24±0.36	0.58±0.40	2.19±4.16
NATR	13	25.06±15.24	14	0.15±0.39	1.03±1.02	2.11±3.18
WTRA	7	48.51±63.80	7	0.02±0.04	1.90±3.49	0.00±0.00
SATL	21	7.32±20.01	24	4.64±12.61	6.96±18.12	21.42±59.95
CARB	3	47.00±46.67	5	0.28±0.62	1.35±1.90	2.23±1.97
ISSG	19	0.92±1.33	19	0.15±0.37	0.61±0.82	25.14±98.58
SPSG	9	23.82±32.98	9	0.33±0.41	1.84±1.40	0.21±0.33
PEQD	6	0.17±0.23	6	0.00±0.00	1.48±0.94	0.05±0.13
PNEC	12	2.80±2.22	12	0.00±0.00	1.92±3.09	0.00±0.01
NPTG	15	0.93±0.70	14	0.00±0.00	0.33±0.52	0.55±1.82

Averaged photic layer depth-integrated abundance of *Trichodesmium* spp. trichomes, *Richelia* colonies, *Rhizosolenia* spp. and *Hemiliaulus hauckii* spp. computed for the tropical and subtropical biogeographical provinces crossed during the Malaspina expedition (Prov.): NASE (NE Atlantic Subtropical Gyral), NATR (North Atlantic Tropical Gyral), WTRA (Western Tropical Atlantic), SATL (South Atlantic Gyral), CARB (Caribbean), SPSG (South Pacific Subtropical Gyre), PEQD (Pacific Equatorial Divergence), PNEC (North Pacific Equatorial Countercurrent) and NPTG (North Pacific Tropical Gyre). *n*_Trich_ and *n*_Dia_ are the number of stations where *Trichodesmium* spp. and the other diazotrophic microphytoplankton groups were determined, respetively. Errors correspond to standard deviations.
