# Supplementary material for: Importance of salt fingering for new nitrogen supply in the oligotrophic ocean
Source: Nat Commun. 2015 Sep 9;6:8002. doi: 10.1038/ncomms9002 (PMC4579400; doi:10.1038/ncomms9002)
Supplement: Supplementary Information — Supplementary Figures 1-2 and Supplementary Table 1 [file ncomms9002-s1.pdf]

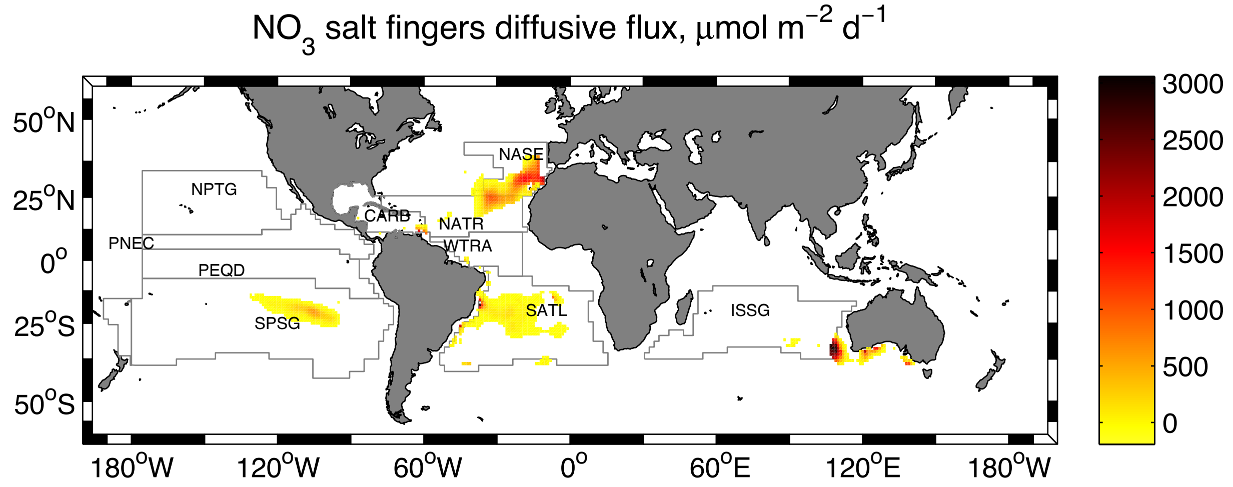

Supplementary Figure 1: Nitrate supply due to salt fingers diffusivity in the global ocean. Vertical nitrate fluxes due to salt fingers diffusivity computed for the global ocean from the World Ocean Atlas 2009 data and the K-Profile Parameterization. White areas indicate regions where no salt fingers' favourable stratification was found at the nitracline depth, and hence salt fingers diffusivity was zero. Main tropical and subtropical biogeographical provinces[14] crossed during the expedition are shown: NASE (NE Atlantic Subtropical Gyral), NATR (North Atlantic Tropical Gyral), WTRA (Western Tropical Atlantic), SATL (South Atlantic Gyral) and CARB (Caribbean) in the Atlantic; ISSG (Indian South Subtropical Gyre) in the Indian and SPSG (South Pacific Subtropical Gyre), PEQD (Pacific Equatorial Divergence), PNEC (North Pacific Equatorial Countercurrent) and NPTG (North Pacific Tropical Gyre) in the Pacific Oceans.

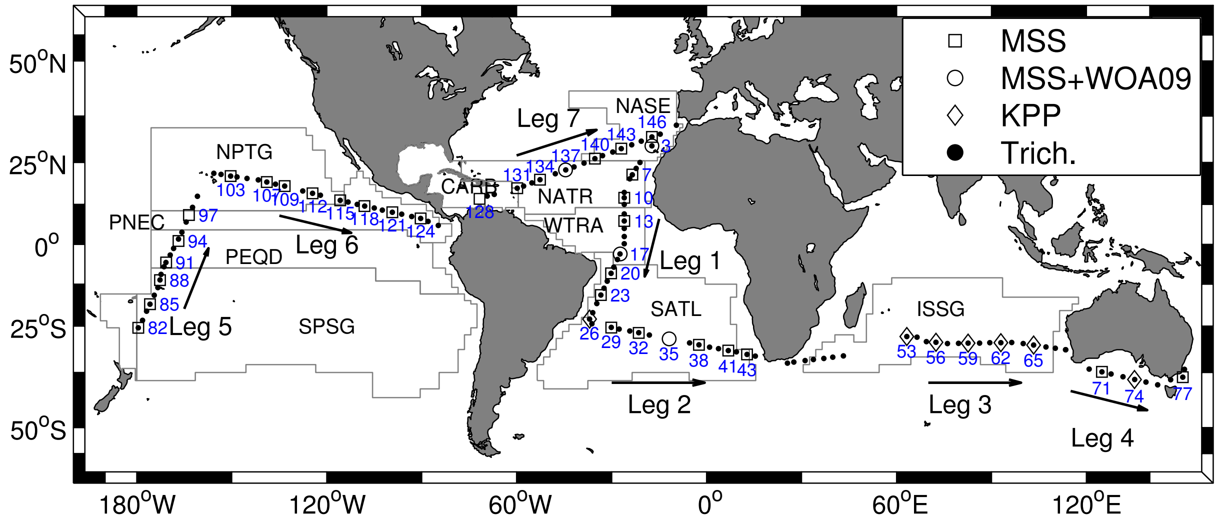

Supplementary Figure 2: Track of the Malaspina circumnavegation expedition. Details of the sampling stations carried out during the Malaspina circumnavegation expedition. Diffusivity was estimated by using a microstructure profiler (MSS, white squares □ and circles ∘) and the K-profile parameterization (KPP, white diamonds ◇). In four stations nitrate concentrations were obtained from the World Ocean Atlas 2009 (WOA09, white circles ∘). Small dots (•) indicate stations sampled for *Trichodesmium* spp. abundance. Numbers indicate station numbers. Main tropical and subtropical biogeographical provinces[14] crossed during the expedition are shown: NASE (NE Atlantic Subtropical Gyral), NATR (North Atlantic Tropical Gyral), WTRA (Western Tropical Atlantic), SATL (South Atlantic Gyral) and CARB (Caribbean) in the Atlantic; ISSG (Indian South Subtropical Gyre) in the Indian and SPSG (South Pacific Subtropical Gyre), PEQD (Pacific Equatorial Divergence), PNEC (North Pacific Equatorial Countercurrent) and NPTG (North Pacific Tropical Gyre) in the Pacific Oceans.

Supplementary Table 1: Contribution of N_2 fixation and nitrate diffusive fluxes to the new nitrogen supply during the Malaspina expedition. Photic layer depth-integrated N_2 fixation rates (N_2 fix.), *Trichodesmium* spp. abundance in the upper 200 m (Trich abun.), nitrate gradient (∂ [NO_3]/ ∂z), vertical diffusivity (K_{t+sf}) and nitrate diffusive fluxes ($Flux_{t+sf}$ NO_3) due to salt fingers plus mechanical turbulence, vertical diffusivity (K_{sf}) and nitrate diffusive fluxes ($Flux_{sf}$ NO_3) due to salt fingers, and relative contribution of N_2 fixation (% N_2 fix.) and salt fingers (% sf.) to the new nitrogen supply, computed for the stations sampled during the Malaspina expedition. L. stands for leg, St. for station and Prov. for the biogeographical province[14] where each station was sampled: NASE (NE Atlantic Subtropical Gyral), NATR (North Atlantic Tropical Gyral), WTRA (Western Tropical Atlantic), SATL (South Atlantic Gyral) and CARB (Caribbean) in the Atlantic; ISSG (Indian South Subtropical Gyre) in the Indian and SPSG (South Pacific Subtropical Gyre), PEQD (Pacific Equatorial Divergence), PNEC (North Pacific Equatorial Countercurrent) and NPTG (North Pacific Tropical Gyre) in the Pacific Oceans. Four stations were sampled along the coastal regions of Australia (SSTC, South Subropical Convergence, AUSW, West Australia Coastal, and AUSE, East Australia Coastal). Errors calculation is explained in the Methods section.

L	St.	Prov.	N_2 fix.	Trich. Abun.	∂ [NO_3]/ ∂z	K_{t+sf}	$Flux_{t+sf}$ NO_3	K_{sf}	$Flux_{sf}$ NO_3	% sf.	% N_2 fix.
				$10^6\cdot$		$10^{-4}\cdot$		$10^{-4}\cdot$			
			µmol m^{-2} d^{-1}	Trich m^{-2}	µmol m^{-4}	(m^2 s^{-1})	µmol m^{-2} d^{-1}	(m^2 s^{-1})	µmol m^{-2} d^{-1}		
	3	NASE	2.9	0.0	-59±10	0.65±0.09	334.1±73.3	0.15±0.06	75.2±32.7	22.32	0.86
	7	NATR	3.9	19.8	-277±58	0.05±0.01	111.8±27.4	0.00±0.00	10.8±10.1	9.31	3.39
	10	NATR	5.0	8.6	-340±39	0.03±0.00	99.3±14.4	0.00±0.00	8.2±5.4	7.82	4.78
1	13	WTRA	8.3	55.2	-357±110	0.05±0.01	144.4±50.9	0.00±0.00	0.0	0.00	5.41
	17	WTRA	2.8	6.2	-262±23	0.37±0.06	846.5±152.1	0.14±0.06	325.6±129.4	38.33	0.33
	20	SATL	4.3	0.2	-293±18	0.09±0.01	221.7±34.3	0.00±0.00	0.0	0.00	1.88
	23	SATL	43.8	3.8	-9±4	0.07±0.01	5.4±2.5	0.03±0.01	2.3±1.2	4.57	88.94
	26	SATL	27.9	0.4	-13±2	1.17±0.43	134.3±52.8	1.07±0.42	122.7±51.2	75.65	17.22
	29	SATL	24.2	1.2	-20±3	0.23±0.02	40.6±6.9	0.10±0.02	17.4±4.3	26.93	37.32
	32	SATL	32.8	2.0	-56±7	0.28±0.03	133.3±22.1	0.13±0.03	63.4±16.3	38.14	19.76
	35	SATL	12.5	-	-52±12	0.33±0.05	149.4±39.3	0.20±0.05	91.4±29.8	56.50	7.70
2	38	SATL	4.0	0.2	-29±4	0.17±0.02	43.5±7.1	0.02±0.01	6.3±3.2	13.22	8.42
	41	SATL	6.0	0.2	-118±21	0.70±0.16	717.0±207.3	0.01±0.01	11.1±7.6	1.53	0.82
	43	SATL	4.9	9.4	-173±45	0.10±0.01	154.6±43.8	0.00±0.00	0.0	0.00	3.09
	53	ISSG	10.0	0.6	-27±8	0.10±0.00	23.1±6.9	0.00±0.00	0.0	0.00	30.09
	56	ISSG	14.4	0.2	-102±9	0.10±0.00	88.8±7.7	0.00±0.00	0.0	0.00	13.97
4	59	ISSG	3.4	0.0	-94±21	0.10±0.00	81.7±18.1	0.00±0.00	0.0	0.00	4.00
	62	ISSG	7.7	0.6	-46±9	0.29±0.17	114.8±72.0	0.19±0.17	74.7±70.3	60.94	6.32
	65	ISSG	7.8	0.4	-76±12	0.14±0.03	94.2±22.8	0.04±0.03	27.7±18.7	27.13	7.65
	67	AUSW	59.5	0.2	-	-	-	-	-	-	-
4	71	SSTC	4.5	0.0	-52±3	7.22±6.00	3231.9±2689.9	0.19±0.05	86.0±22.7	2.66	0.14
	74	SSTC	3.7	0.4	-71±7	25.62±0.31	15823.4±1571.9	0.33±0.32	201.4±200.2	1.27	0.02
	77	AUSE	4.3	13.0	-110±18	0.08±0.01	76.2±15.7	0.00±0.00	0.0	0.00	5.39
	82	SPSG	1.6±1.6	0.4	-23±2	0.45±0.06	89.4±14.4	0.00±0.00	0.0	0.00	1.79
	85	SPSG	14.2±1.7	76.6	-24±4	0.26±0.02	53.7±9.6	0.00±0.00	0.0	0.00	20.96
5	88	SPSG	0.1±0.3	1.2	-66±7	0.08±0.02	47.1±12.3	0.00±0.00	0.0	0.00	0.29
	91	PEQD	0.0±0.3	0.2	-118±13	0.11±0.02	114.3±24.5	0.00±0.00	0.0	0.00	0.03
	94	PEQD	0.5±0.3	0.0	-156±12	0.08±0.01	110.1±19.8	0.00±0.00	0.0	0.00	0.45
	97	PNEC	0.2±0.2	-	-303±34	0.19±0.05	488.7±135.2	0.00±0.00	0.0	0.00	0.04
	103	NPTG	14.3	2.4	-84±26	0.67±0.23	483.3±223.4	0.09±0.06	64.5±44.6	12.96	2.88
	107	NPTG	4.7	0.8	-33±1	0.05±0.01	15.2±2.0	0.01±0.00	1.9±1.2	9.73	23.63
	109	NPTG	18.0	0.0	-209±41	0.06±0.01	113.0±27.6	0.00±0.00	0.0	0.00	13.74
6	112	NPTG	2.3	0.2	-157±16	0.05±0.00	61.4±8.2	0.00±0.00	0.0	0.00	3.66
	115	NPTG	7.0	0.8	-225±75	0.05±0.01	94.9±33.2	0.00±0.00	0.0	0.00	6.92
	118	PNEC	4.5	2.4	-223±71	0.16±0.05	314.2±132.5	0.00±0.00	0.0	0.00	1.43
	121	PNEC	1.8	0.6	-292±56	0.10±0.04	251.8±117.3	0.00±0.00	0.0	0.00	0.70
	124	PNEC	1.3	2.2	-382±43	0.16±0.06	534.2±214.0	0.00±0.00	0.0	0.00	0.24
	128	CARB	7.7	-	-120±4	0.03±0.00	30.6±2.0	0.00±0.00	3.3±1.3	8.65	20.18
	131	CARB	4.4	25.0	-41±6	0.06±0.01	20.7±3.9	0.00±0.00	0.0	0.00	17.48
	134	NATR	18.3	24.0	-46±2	0.24±0.04	96.4±15.4	0.00±0.00	0.5±0.3	0.45	15.92
7	137	NATR	7.7	24.6	-44±3	0.24±0.03	92.2±14.3	0.02±0.01	7.0±4.6	7.02	7.72
	140	NASE	10.2	18.4	-61±3	0.15±0.01	81.5±8.7	0.03±0.01	16.2±4.7	17.63	11.17
	143	NASE	6.5	1.2	-49±3	0.27±0.02	113.7±11.5	0.06±0.02	27.1±9.4	22.52	5.44
	146	NASE	7.7	2.0	-38±2	0.34±0.02	110.8±7.8	0.05±0.01	16.8±4.7	14.18	6.53
